# Public support for health taxes and media regulation of harmful products in South Korea

**DOI:** 10.1186/s12889-019-7044-2

**Published:** 2019-05-30

**Authors:** Kyae Hyung Kim, EunKyo Kang, Young Ho Yun

**Affiliations:** 1 Department of Family Medicine, and Institute for Public Health and Medical Service, Seoul National University Hospital, Seoul, South Korea; 2 Department of Family Medicine and Department of Biomedical Science, Seoul National University College of Medicine, 103 Daehak-ro, Jongno-gu, Seoul, 110-799 South Korea

**Keywords:** Food tax, Perception, Smoking, Alcohol, Public support

## Abstract

**Background:**

Public health policy is inevitably associated with either a strong presence or lack of public support. We investigated factors associated with both the public support of and opposition to health taxes and the media regulation regarding advertising harmful products in Korea.

**Methods:**

We interviewed 1200 respondents that were recruited using an equal-probability sampling method in accordance with the 2016 Korean census. Our investigation examined the extent of support and opposition towards health taxes and the media regulation of advertising that targets the consumption of tobacco, alcohol, and unhealthy foods according to socioeconomic characteristics, health habits, body mass index (BMI), and exposure to the advertising of harmful products. The study was conducted using a univariate and stepwise multivariate regression analysis.

**Results:**

The majority (71.8%) of the respondents were supportive of imposing health taxes in general. Despite a high prevalence of tobacco and alcohol consumption among the respondents, they strongly supported media regulation of tobacco (72.3%), alcohol (63.7%), and eating broadcasts (51.9%) food advertising (44.0%). Those that were non-smokers, earned a high-income, were married, or had a child were likely to support at least one kind of regulation regarding alcohol and smoking related advertising. An exposure to excessive advertising of unhealthy products was associated with increase of respondents supporting the media regulation. Those who regarded the media as being influential seemed to be more supportive of health taxes or media regulation.

**Conclusion:**

Our results indicated strong public support among the respondents for health taxes and the media regulation regarding the advertising of unhealthy products. Based on our data, we are optimistic that countries whose population show a high rate of tobacco, alcohol or unhealthy food consumption may launch public policy in addressing these factors.

**Electronic supplementary material:**

The online version of this article (10.1186/s12889-019-7044-2) contains supplementary material, which is available to authorized users.

## Background

Managing risk factors for chronic diseases has long been an important global issue. The prevalence of obesity has doubled in 73 countries since 1980, and cardiovascular disease has become the leading cause of death and of disability [1]. Dietary risk factors and low physical activity accounted for 10.0% of deaths and disabilities globally [2]. In addition, tobacco smoking (including secondhand smoke) and alcohol consumption were leading attributable risks, accounting for 6.3 and 4.8%, respectively, of deaths and disabilities worldwide [2]. Alcohol consumption causes more than 200 diseases and injuries resulting in death and disability relatively early in life [3]. In the age group of 20 to 39, about 13.5% of total deaths are due to alcohol [3]. In South Korea, the prevalence of obesity, tobacco use, and alcohol abuse is notably high when compared to other OECD countries [4–6].

Taxes, subsidies, and welfare policies on unhealthy and harmful products have known to be more effective than information campaigns on reducing their consumption [7]. Taxes have been used in many countries to deter smoking and harmful alcohol use, and more recently, an increasing number of governments have sought to expand this approach to foods and beverages that are high in fat, salt, or sugar [8]. In general, governments tend to be very cautious about introducing new taxes or raising existing taxes, despite the significant benefits that health taxes can produce. Taxes are never popular, and the burden of taxation on tobacco, alcohol products, foods, and beverages tends to be larger for low-income consumers [7, 9]. Cross-border and illicit trade are major negative phenomena, especially for alcohol and tobacco products [10]. Despite public reluctance to tax, earmarking taxes is a way to gain public support for imposing taxes. As stated by the WHO report, about 30 countries have earmarked tobacco tax revenues for health purposes, which has given positive public supports for tobacco tax [11]. In Korea, cigarette prices almost doubled due to taxation in early 2015, [12] while commodities such as alcohol, sugar-sweetened beverages (SSB), and unhealthy, Energy Dense Nutrition Poor (EDNP) foods are still not subject to health-related taxation.

Unhealthy media environment is also shown to be a key driver of chronic diseases [13]. Smokers and alcohol drinkers are often depicted as being more successful and healthier in movies than they are in real life. Product placement of alcohol products and brands is very common in movies, and reality-shows in Korea. Exposure to food and beverage advertising on television and online [14] on YouTube, [15] Facebook, [16] and other social media, [17] has been shown to influence the food preferences, purchases, and consumption of these products, especially pertaining to youths. Eating broadcasts (Foodporn, Mukbang in Korean [18]) that have a strong presence in social media rely on close-up images of cooking, provocatively presented foods that are high in fat, salt and sugar, as well as exotic dishes, soft drinks, liqueurs, etc. Advertising and marketing professionals have started using the eating broadcast trend, which is very popular among individuals in their 20’s and 30’s, as a new advertising approach [19, 20]. In response, regulations of the marketing of the eating broadcasts through the mass media has been suggested.

Public health policy has inevitably needed public support [21]. We investigated factors associated with both the public support of and opposition to health taxes and regulation in a sampled population in South Korea.

## Methods

### Participants and procedures

This survey was conducted from April 4 to May 7, 2018. Interviewees were recruited using an equal-probability sampling method according to the 2016 Korean census, taking the age and sex strata by region. Eligible participants fulfilled the following criteria: 1) 19 years of age or older, 2) able to understand the purpose of the study, and 3) able to read and answer the questionnaires in Korean. Participants were informed of the scope of the study by trained research assistants at the beginning of the study, and completed a semi-structured, self-reported questionnaire paper at the completion. The study was carried out by World Research Co., Ltd., and 1200 participants completed the questionnaire (response rate was approximately 30%).

### Measurement

#### Opinions on alcohol advertisement, smoking scenes portrayed in the media, food-shows, and food advertisement regulations

Participants received questions regarding their opinions on the negative impact of alcohol advertising, smoking scenes portrayed by the mass media, eating broadcasts, and food advertising on health habits (Additional file 1). The respondents replied to the question, “Some believe that regulatory measures are necessary in the following areas as their portrayal in the mass media has a negative impact on viewers’ health habits. Do you think it is necessary to regulate these areas?” with a range of 1 to 4 (1 = strongly disagree, 2 = disagree, 3 = agree, 4 = strongly agree). The areas presented were: 1) alcohol advertising, 2) smoking scenes portrayed by the mass media, 3) eating broadcasts, and 4) food advertisement. The definition of eating broadcasts that was provided was “Broadcasting that shows host eating and communicating online and via ‘Afreeca TV’ (example of internet/social media-based TV).”

#### Exposure to alcohol advertising, smoking scenes portrayed by the media, eating broadcasts, and food advertising and their influence on health habits

To determine the participants’ degree of exposure to alcohol advertising, smoking scenes portrayed by the media, eating broadcasts, and food advertising, respondents were asked the following question during the last week: “How often have you been exposed to the following factors during the last week?”. Participants selected one of four answers: 1) Never, 2) Rarely, 3) Occasionally, and 4) Always. And how these four factors would affect their health habits, respondents were asked the following question during the last week: “How do the following factors affect your health habits?” Their responses were based on a 4-point scale: 1) not at all affected 2) mostly not affected, 3) mostly affected, and 4) strongly affected.

#### Health tax

The World Health Organization (WHO) recommended introducing a sugar tax in 2016 on sugar-sweetened beverages (SSB) containing sugars known to be a major cause of chronic diseases [22]. In this regard, we asked respondents how they felt about imposing health taxes, such as environmental or tobacco consumption taxes, on companies. Interviewees responded using a 4-point Likert scale that ranged from “strongly disagree” to “strongly agree.”

#### Sociodemographic variables

We collected the respondents’ sociodemographic information included age, sex, marital status, educational status, monthly income, job status, and household composition. Health related variables such as BMI, smoking, and drinking were also assessed.

### Statistical analysis

In this study, we analyzed factors that are related to how individuals perceive health taxes, as well as alcohol advertising, smoking scenes portrayed by the media, eating broadcasts, and food advertising regulations, and conducted univariate analyses to measure the relationship between the respondents’ views on these items, as well as sociodemographic variables, and health related factors. We also performed univariate analyses to assess the association between the exposure to these factors, their influence on the individuals, as well as the resulting views the individuals have on regulation. Socio-demographic factors, health-related variables, and exposure and influence factors that were found to be significant in univariate analysis were used to investigate associations related to the individuals’ views on regulation. In the final model, we performed multivariate regression analysis to identify independent factors that were statistically significant. The statistical tests performed included the Pearson’s chi-square for comparison of proportions. In all analyses, we determined two-sided *p*-values and considered a p-value that was less than 0.05 to be significant. All data were analyzed with SPSS software version 23.0 (Armonk, NY, USA) and R software version 3.5.1 in RStudio® version 1.1.456 (RStudio Inc., Boston, MA, USA).

## Results

### Demographic characteristics of study participants

Demographics and clinical characteristics for the 1200 respondents of the study are provided in Table 1. The average age of the participants was 46.97 years, and more than half were female (50.7%). Most of them were married or living with a partner (73.7%). High school graduates and higher education graduates showed similar characteristics. Most individuals had a monthly income between $3000 and $6000, and 70.0% were employed. 74.6% of respondents had one or more children, most were non-smokers, and drank less than once a week.

**Table1:** Demographics and Clinical Characteristics of Participants (*N* = 1200)

Characteristic		No	%
Age	Mean + SD	46.97 ± 14.18	
Sex	Male	592	49.3
	Female	608	50.7
Marital status	≤ Not married	265	22.1
	Divorced/Widowed/Separated	51	4.3
	Married/Cohabited	884	73.7
Education	≤Middle school graduate	124	10.3
	High school	537	44.8
	≥College or University	539	44.9
Income	< 3000$	307	25.7
	3000$ - 6000$	809	67.8
	> 6000$	78	6.5
Job status	Gainfully employed	840	70.0
	Dependents	360	30.0
Household composition	No children	305	25.4
	One or more children	895	74.6
Smoking status	Current Smoker	270	22.5
	Ex-smoker	144	12.0
	Non-smoker	786	65.5
Drinking	> 1 time per week	414	34.5
	≤ 1 time per week	786	65.5
BMI	Underweight	41	3.4
	Normal weight	591	49.3
	Overweight	344	28.7
	Obese	224	18.7
Alcohol Advertisement	Agree with regulation	764	63.7
	Against regulation	436	36.3
Smoking scene	Agree with regulation	868	72.3
	Against regulation	332	27.7
Eating broadcasts	Agree with regulation	623	51.9
	Against regulation	577	48.1
Food Advertisement	Agree with regulation	529	44.1
	Against regulation	671	55.9
Health Tax	Agree to impose	861	71.8
	Disagree to impose	339	28.2

Abbreviation: SD, Standard Deviation

### Opinions about mass media regulations and health taxes

Regarding alcohol advertising and smoking scenes portrayed by the media, more than half of the respondents were in favor of their regulation (63.7 and 72.3%, respectively). 51.9% of the respondents were in favor of the regulation of eating broadcasts, and 44.1% for the regulation of food advertising. 71.8% of the participants indicated that they agreed with introducing health taxes.

### Relationship between sociodemographic variables and perception of health taxes and mass media regulation

Regarding the advertising of alcohol, married respondents were more in favor of regulation than unmarried respondents, while respondents who had at least one child indicated a higher level of approval than those who had no children (Table 2). Women were more likely to be in favor of regulations on smoking than men, and graduates of higher education more than graduates of middle school or lower. Supporters of smoking regulations tended to have higher incomes, and in the case of eating broadcasts, individuals who were married, divorced, widowed, and had children tended to support regulations in comparison to those who were unmarried. We found no indication of a sociodemographic factor correlating with being in favor of the regulation of food advertising. Respondents aged 40 and over, as well as those who were married, divorced, or separated were more in favor of imposing health taxes. While individuals who had jobs and those who had children were more likely to agree on imposing health taxes, those with a monthly income of more than $6000 were less likely to be in favor of health taxes.

**Table 2:** Univariate associations between sociodemographic variables and perception of health tax and mass media regulation

	Regulation								Health Tax	
	Alcohol advertising		Smoking scene		Eating broadcasts		Food advertising			
	OR (95% CI)	*p*-value	OR (95% CI)	*p*-value	OR (95% CI)	*p*-value	OR (95% CI)	*p*-value	OR (95% CI)	*p*-value
Age										
20–39	1 (Ref)		1 (Ref)		1 (Ref)		1 (Ref)		1 (Ref)	
≥ 40	1.15 (0.89–1.47)	N/S	0.86 (0.65–1.12)	N/S	1.19 (0.94–1.52)	N/S	0.97 (0.76–1.23)	N/S	1.37 (1.05–1.77)	0.019
Sex										
Male	1 (Ref)		1 (Ref)		1 (Ref)		1 (Ref)		1 (Ref)	
Female	1.15 (0.91–1.46)	N/S	1.43 (1.11–1.84)	0.006	1.07 (0.86–1.35)	N/S	1.04 (0.83–1.31)	N/S	1.00 (0.78–1.28)	N/S
Marital Status										
Not married	1 (Ref)		1 (Ref)		1 (Ref)		1 (Ref)		1 (Ref)	
Divorced/Widowed/Separated	1.31 (1.00–1.74)	0.048	1.01 (0.74–1.37)	N/S	1.35 (1.03–1.78)	0.032	1.18 (0.89–1.56)	N/S	1.59 (1.19–2.13)	0.002
Married/Cohabited	1.55 (0.82–2.94)	N/S	1.26 (0.63–2.54)	N/S	2.00 (1.08–3.72)	0.027	1.40 (0.77–2.55)	N/S	2.33 (1.12–4.86)	0.024
Educational Status										
≤ Middle school	1 (Ref)		1 (Ref)		1 (Ref)		1 (Ref)		1 (Ref)	
High school	1.34 (0.90–2.00)	N/S	1.45 (0.96–2.19)	N/S	1.03 (0.70–1.52)	N/S	1.05 (0.71–1.56)	N/S	0.75 (0.47–1.18)	N/S
College or higher	1.26 (0.85–1.88)	N/S	1.57 (1.04–2.37)	0.034	1.00 (0.68–1.48)	N/S	1.15 (0.78–1.71)	N/S	0.76 (0.48–1.20)	N/S
Monthly Income										
< 3000$	1 (Ref)		1 (Ref)		1 (Ref)		1 (Ref)		1 (Ref)	
3000$ - 6000$	1.16 (0.89–1.53)	N/S	1.41 (1.06–1.88)	0.017	1.07 (0.82–1.39)	N/S	0.91 (0.70–1.18)	N/S	1.00 (0.75–1.35)	N/S
> 6000$	1.01 (0.61–1.69)	N/S	1.96 (1.08–3.56)	0.028	0.86 (0.52–1.42)	N/S	0.73 (0.44–1.21)	N/S	0.51 (0.31–0.86)	0.011
Job Status										
No	1 (Ref)		1 (Ref)		1 (Ref)		1 (Ref)		1 (Ref)	
Yes	1.18 (0.90–1.56)	N/S	1.14 (0.84–1.55)	N/S	1.21 (0.92–1.58)	N/S	1.21 (0.92–1.59)	N/S	1.43 (1.07–1.92)	0.017
Household composition										
No children	1 (Ref)		1 (Ref)		1 (Ref)		1 (Ref)		1 (Ref)	
One or more children	1.40 (1.08–1.83)	0.012	1.08 (0.81–1.44)	N/S	1.34 (1.06–1.61)	0.014	1.08 (0.83–1.41)	N/S	1.51 (1.15–2.00)	0.004

Abbreviations: *OR* odds ratio, *Ref* reference, *N/S* Non-significant

### Univariate analysis of perception of health taxes and mass media regulation in regards to smoking, drinking, and BMI status

In Table 3, the opinions about the regulation of alcohol advertising showed a relationship between an individual’s smoking status and their BMI. Ex-smokers and non-smokers tended to prefer these regulations, while underweight individuals showed a tendency to see alcohol advertising regulations less favorably. Regarding the regulation of smoking scenes portrayed by the media, non-smokers tended to be more in favor of the regulations. In the case of eating broadcasts, underweight respondents tended to disagree with their regulation, and non-smokers favored the regulation of food advertising. There was no relationship between the consumption of alcohol, BMI, and health taxes, but ex-smokers and non-smokers showed a tendency to favor health taxes.

**Table 3:** Univariate analysis of perception of health tax and mass media regulation with smoking, drinking and BMI status

	Regulation												Health Tax		
	Alcohol advertising			Smoking scene			Eating broadcasts			Food advertising					
	*N* (%)	OR (95% CI)	*p*-value	*N* (%)	OR (95% CI)	*p*-value	*N* (%)	OR (95% CI)	*p*-value	*N* (%)	OR (95% CI)	*p*-value	N (%)	OR (95% CI)	*p*-value
Smoking															
Current smoker	156 (57.8)	1 (Ref)		183 (67.8)	1 (Ref)		131 (48.5)	1 (Ref)		103 (38.1)	1 (Ref)		178 (65.9)	1 (Ref)	
Ex-smoker	98 (68.1)	1.56 (1.02–2.38)	0.041	99 (68.8)	1.05 (0.68–1.62)	N/S	76 (52.8)	1.19 (0.79–1.78)	N/S	64 (44.4)	1.30 (0.86–1.96)	N/S	111 (77.1)	1.74 (1.09–2.76)	0.019
Non-smoker	510 (64.9)	1.35 (1.02–1.79)	0.037	586 (74.6)	1.39 (1.03–1.88)	0.031	416 (52.9)	1.19 (0.91–1.57)	N/S	362 (46.1)	1.38 (1.04–1.84)	0.024	572 (72.8)	1.38 (1.03–1.86)	0.033
Drinking															
> 1 time per week	263 (63.5)	1 (Ref)		296 (71.5)	1 (Ref)		203 (49.0)	1 (Ref)		181 (43.7)	1 (Ref)		297 (71.7)	1 (Ref)	
≤ 1 time per week	501 (63.7)	0.99 (0.77–1.27)	N/S	572 (72.8)	0.94 (0.72–1.22)	N/S	420 (53.4)	0.84 (0.66–1.06)	N/S	348 (44.3)	0.98 (0.77–1.24)	N/S	564 (71.8)	1.00 (0.77–1.30)	N/S
BMI															
Normal weight	374 (63.3)	1 (Ref)		423 (71.6)	1 (Ref)		315 (53.3)	1 (Ref)		263 (44.5)	1 (Ref)		424 (71.7)	1 (Ref)	
Underweight	19 (46.3)	0.50 (0.27–0.95)	0.033	28 (68.3)	0.86 (0.43–1.69)	N/S	14 (34.1)	0.45 (0.23–0.88)	0.020	15 (36.6)	0.72 (0.37–1.39)	N/S	27 (65.9)	0.76 (0.39–1.48)	N/S
Overweight	216 (63.5)	1.02 (0.77–1.34)	N/S	243 (71.5)	1.00 (0.74–1.34)	N/S	175 (51.5)	0.92 (0.70–1.20)	N/S	151 (44.4)	1.00 (0.77–1.31)	N/S	253 (74.4)	1.15 (0.85–1.55)	N/S
Obese	155 (68.0)	1.23 (0.88–1.70)	N/S	174 (76.3)	1.28 (0.90–1.83)	N/S	119 (52.2)	0.98 (0.72–1.33)	N/S	100 (43.9)	0.97 (0.71–1.32)	N/S	157 (68.9)	0.87 (0.62–1.21)	N/S

Abbreviations: *OR* odds ratio, *Ref* reference, *N/S* Non-significant

### Association of mass media exposure and its influence, and perception of health taxes, and mass media regulation

Table 4 showed the association of mass media regulation and mass media exposure and its influence. All those who were exposed or influenced by alcohol advertising were found to be more in favor of its regulation. In the case of smoking scenes portrayed by the media, people who were exposed or influenced by them were more likely to agree with their regulation, and similarly, those who were exposed or influenced by eating broadcasts were more likely to favor their regulation. In the case of food advertising, exposure and influence had a similar impact. Individuals who were exposed to and influenced by alcohol advertising (in this case, exposure to alcohol advertising had no effect, while being influenced by it did have an effect), smoking scenes portrayed by the media, eating broadcasts, and food advertising showed a tendency to favor health taxes.

**Table 4:** Univariate analysis with Support of health tax and mass media regulation according to mass media exposure and influence

	Regulation								Health Tax	
	Alcohol advertising		Smoking scene		Eating broadcasts		Food advertising			
	OR (95% CI)	*p*-value	OR (95% CI)	*p*-value	OR (95% CI)	*p*-value	OR (95% CI)	*p*-value	OR (95% CI)	*p*-value
Exposure to alcohol advertising										
No	1 (Ref)		1 (Ref)		1 (Ref)		1 (Ref)		1 (Ref)	
Yes	2.05 (1.62–2.61)	< 0.001	2.29 (1.77–2.96)	< 0.001	1.45 (1.15–1.82)	0.002	1.35 (1.07–1.70)	0.011	1.26 (0.98–1.62)	N/S
Influenced by alcohol advertising										
No	1 (Ref)		1 (Ref)		1 (Ref)		1 (Ref)		1 (Ref)	
Yes	4.45 (3.41–5.81)	< 0.001	2.79 (2.12–3.68)	< 0.001	2.82 (2.23–3.58)	< 0.001	3.04 (2.39–3.85)	< 0.001	2.04 (1.57–2.65)	< 0.001
Exposure to smoking scene										
No	1 (Ref)		1 (Ref)		1 (Ref)		1 (Ref)		1 (Ref)	
Yes	1.97 (1.52–2.54)	< 0.001	1.98 (1.50–2.63)	< 0.001	2.12 (1.66–2.70)	< 0.001	2.00 (1.57–2.54)	< 0.001	1.98 (1.50–2.62)	< 0.001
Influenced by smoking scene										
No	1 (Ref)		1 (Ref)		1 (Ref)		1 (Ref)		1 (Ref)	
Yes	2.94 (2.26–3.82)	< 0.001	3.20 (2.38–4.30)	< 0.001	2.58 (2.03–3.28)	< 0.001	2.53 (1.99–3.21)	< 0.001	2.28 (1.72–3.00)	< 0.001
Exposure to eating broadcasts										
No	1 (Ref)		1 (Ref)		1 (Ref)		1 (Ref)		1 (Ref)	
Yes	2.24 (1.73–2.89)	< 0.001	2.57 (1.97–3.36)	< 0.001	2.20 (1.71–2.85)	< 0.001	1.39 (1.08–1.79)	0.011	1.69 (1.30–2.21)	< 0.001
Influenced by eating broadcasts										
No	1 (Ref)		1 (Ref)		1 (Ref)		1 (Ref)		1 (Ref)	
Yes	2.07 (1.62–2.64)	< 0.001	1.79 (1.38–2.32)	< 0.001	2.65 (2.08–3.38)	< 0.001	2.08 (1.62–2.66)	< 0.001	1.46 (1.13–1.89)	0.004
Exposure to food advertising										
No	1 (Ref)		1 (Ref)		1 (Ref)		1 (Ref)		1 (Ref)	
Yes	1.53 (1.18–1.99)	0.001	2.47 (1.88–3.24)	< 0.001	1.80 (1.39–2.34)	< 0.001	1.68 (1.29–2.19)	< 0.001	1.77 (1.34–2.32)	< 0.001
Influenced by food advertising										
No	1 (Ref)		1 (Ref)		1 (Ref)		1 (Ref)		1 (Ref)	
Yes	2.15 (1.69–2.74)	< 0.001	1.68 (1.30–2.17)	< 0.001	2.33 (1.84–2.95)	< 0.001	3.48 (2.71–4.48)	< 0.001	1.85 (1.44–2.39)	< 0.001

Abbreviations: *OR* odds ratio, *Ref* reference, *N/S* Non-significant

### Multivariate logistic regression models for factors associated with views of health taxes and mass media regulation

In the case of the regulation of alcohol advertising, independent factors with statistical significance were household composition, influence of alcohol advertising, and exposure to eating broadcasts. The respondents who had a higher income level, one or more children, were non-smokers, had been exposed to alcohol advertising and eating broadcasts, and had been influenced by smoking scenes portrayed by the media, were more likely to agree to the regulation of smoking scenes in the media. All those who were married, divorced, widowed, had been exposed to smoking scenes or eating broadcasts, and influenced by alcohol advertising, smoking scenes in the media, or eating broadcasts indicated a tendency towards supporting the regulation of eating broadcasts. Regarding the regulation of food advertising, participants who were exposed to alcohol advertising or smoking scenes in the media, and influenced by alcohol advertising, or food advertising, were more likely to consent to the regulation of food advertising. In addition, factors such as older age, being married, divorced, or widowed, having a job, having one or more children, being an ex-smoker or non-smoker, having been exposed to smoking scenes in the media, or eating broadcasts, as well as having been influenced by smoking scenes in the media, or food advertising, were associated with having positive views towards the imposition of health taxes. (Table 5).

**Table 5:** Forward Stepwise regression analysis with perception of health tax and mass media regulation^a^

		Regulation								Health Tax	
		Alcohol advertising		Smoking scene		Eating broadcasts		Food advertising			
		aOR (95% CI)	*p*-value	aOR (95% CI)	*p*-value	aOR (95% CI)	*p*-value	aOR (95% CI)	*p*-value	aOR (95% CI)	*p*-value
Age											
20–39		N/S		N/S		N/S		N/S		1 (Ref)	
≥ 40										1.39 (1.01–1.91)	0.042
Marital Status											
Not married		N/S		N/S		1 (Ref)		N/S		1 (Ref)	
Divorced/Widowed/Separated						1.43 (1.07–1.92)	0.016			1.52 (1.12–2.07)	0.007
Married/Cohabited						2.39 (1.23–4.65)	0.010			2.21 (1.03–4.71)	0.041
Monthly Income											
< 3000$		N/S		1 (Ref)		N/S		N/S		N/S	
3000$ - 6000$				1.51 (1.11–2.04)	0.008						
> 6000$				2.29 (1.22–4.30)	0.010						
Job Status											
No		N/S		N/S		N/S		N/S		1 (Ref)	
Yes										1.50 (1.12–2.01)	0.006
Household composition											
No children		1 (Ref)		1 (Ref)		N/S		N/S		1 (Ref)	
One or more children		1.49 (1.12–1.98)	0.006	1.52 (1.16–1.98)	0.003					1.50 (1.15–1.97)	0.004
Smoking											
Current smoker		N/S		1 (Ref)		N/S		N/S		1 (Ref)	
Ex-smoker				1.07 (0.67–1.70)	N/S					1.55 (0.96–2.52)	N/S
Non-smoker				1.52 (1.10–2.09)	0.011					1.55 (1.12–2.14)	0.008
Exposure to alcohol advertising											
No		N/S		1 (Ref)		N/S		1 (Ref)		N/S	
Yes				1.60 (1.21–2.13)	0.001			1.52 (1.14–2.03)	0.004		
Influenced by alcohol advertising											
No		1 (Ref)		N/S		1 (Ref)		1 (Ref)		N/S	
Yes		4.01 (3.06–5.25)	< 0.001			1.80 (1.34–2.44)	< 0.001	2.32 (1.76–3.06)	< 0.001		
Exposure to smoking scene											
No		N/S		N/S		1 (Ref)		1 (Ref)		1 (Ref)	
Yes						1.34 (1.02–1.75)	0.037	1.53 (1.16–2.03)	0.003	1.49 (1.09–2.02)	0.012
Influenced by smoking scene											
No		N/S		1 (Ref)		1 (Ref)		N/S		1 (Ref)	
Yes				2.79 (2.05–3.81)	< 0.001	1.40 (1.02–1.91)	0.036			1.77 (1.30–2.40)	< 0.001
Exposure to eating broadcasts											
No		1 (Ref)		1 (Ref)		1 (Ref)		N/S		1 (Ref)	
Yes		1.74 (1.33–2.28)	< 0.001	1.96 (1.47–2.62)	< 0.001	1.41 (1.05–1.90)	0.022			1.34 (1.00–1.79)	0.047
Influenced by eating broadcasts											
No		N/S		N/S		1 (Ref)		N/S		N/S	
Yes						1.74 (1.31–2.32)	< 0.001				
Influenced by food advertising											
No		N/S		N/S		N/S		1 (Ref)		1 (Ref)	
Yes								2.72 (2.08–3.57)	< 0.001	1.35 (1.02–1.79)	0.036

Abbreviations: *aOR* adjusted odds ratio, *Ref* reference, *N/S* Non-significant

^a^Multiple logistic regression analysis including variables identified as independent predictors that showed statistical significance in univariate analysis

## Discussion

The present study shows that there is a strong public support for strengthening health policies in Korea. Around 70% of the study sample was supportive of imposing health taxes in general. It was a high proportion considering a previous study from 2012 conducted in 27 European countries on tobacco taxes, ranging from 38.4% (Greece) to 71.3% (Finland) of respondents indicating their support of the taxes [23]. Public support for health taxes in Korea was higher than that in 2014 for SSB taxes in the United States, which only indicated 40.0% [24] and 57.7% in France in 2012 [25]. Regarding the advertising of unhealthy products, there was a strong support amongst Koreans, with the highest support being for the regulation of tobacco (72.3%), alcohol (63.7%), and food/sugar (40–52%), which was consistent with previous studies [25–27]. Among East Asian countries, there is scarce data regarding public support in this area. Korea has a similar smoking rate (22.5%) to China (23.3%) and Japan, (21.7%) but has a higher level of tobacco regulation due to the government’s strong will and public support. While most countries that have high rates of tobacco, alcohol, and obesogenic-food consumption are cautious in introducing regulations and tax policies, our data on public support are promising.

Individual factors such as age, sex, and education level can have an influence on the support of public health policies [25, 28]. McMillen et al. reported that older populations in the US showed higher levels of supporting regulatory tobacco policies, [28] and women to be more likely to support both regulatory policies on tobacco [28] and alcohol, [29] but opposing regulatory SSB policies. [25] In some studies, the higher the level of education, the higher the degree of support [25, 30]. However, in this study, the multivariate model showed that public support and opposition to regulations were not related to age, sex, or education. In another study, high-income urban Koreans seemed to consume more SSB than others [31]. Even highly educated Korean men consumed higher rates of tobacco and alcohol, as they are regarded as a way to promote social ties in competitive workplaces and military service [6, 32].

In our study, non-smokers are more supportive of media regulation and health taxes, which is consistent with previous studies [4–6, 33]. Alcohol consumption and BMI show no correlation with being for or against taxes and regulation. Individuals who were married or living with a partner were more likely to support the regulation of eating broadcasts and taxes on unhealthy products. Individuals with one or more children also showed support for regulating alcohol advertising and smoking scenes in the media. In regards to these results, we find it reasonable to build a supportive base for regulation and taxes through family-based advocacy and by extending children- and youth-centered policies to the rest of the population.

In the present study, exposure to the advertising of unhealthy products resulted in a tendency towards support for its regulation, which was clearly not what industries and advertising companies intended. Meyvis et al. described that irrelevant information systematically weakens the consumers’ expectations that the product will provide the promised benefit [34]. In Korea, alcohol advertising exposure in 2016 included terrestrial TV (32,313 times in 2016), cable TV (277,769 times), and general service TV (including IPTV, satellite TV, 2897 times) which resulted in 890 instances of exposure per day [35]. From our perspective, this indicates that the public is not responding positively to alcohol advertising. Children and adolescents whose judgement abilities are not yet fully developed are most susceptible to the influences of such advertising [14]. In addition to the exposure to alcohol advertising, exposure to food advertising increases food intake, BMI, body fat percentage, and trunk adiposity, especially in children and adolescent [36, 37]. Regulations and subsidies that encourage companies to produce healthier products and advertise health benefits would be effective and create healthier environments [38].

Regulatory tobacco interventions are underway in Korea, such as the introduction of the national legal minimum age and the regulation of off-premise sales and smoking bans in public places that have been in place since 2016 [12]. Warning messages on cigarette packs increased in size and were made more graphic starting in 2015 [12]. Prices of cigarettes almost doubled due to taxation in early 2015 [12]. Alcohol regulation has taken its first steps towards enforcement, with strong public support, banning advertisements by models drinking alcohol and placement of beer gulping sound effects to entice viewers starting as early as 2020.

Over the past several years, Korea’s obesity-related policies have been focused on youths, which entails a ban on offering SSB in schools. EDNP foods advertising are currently unavailable on TV between 5 pm and 7 pm, [39] and recently, the government has begun to expand its anti-obesity policies for the entire population, which includes the regulation of TV eating broadcasts. After realizing that viewers were interested in ‘the act of eating itself,’ food companies began to engage in marketing activities in which entertainers and movie stars consume their products on TV for public broadcasting [19]. For example, when a famous female singer ate ‘Gopchang (Grilled Beef Tripe)’ in her documentary show, the total sales of it increased 600% in 2018 [40]. However, regulation remains controversial as claims have been made that it violates the basic rights of the people. Likewise, taxes on sugar, salt, or fat are generally regarded as being unlikely in the near future in Korea, although taxes that increase their prices by 20% or more could reduce the consumption of unhealthy products [41]. Government policies had continued to support the growth of agricultural production, food industries, and distributors spurred by existing perspectives on food security, economics, and trades. However, the recent role of the government has changed to coordinate multidimensional and multi-faceted strategies to improve the population’s health. There should be a greater emphasis on strengthening a supportive base through family-centered advocacy and broaden its reach to the entire population by further regulations and taxes on alcohol, SSB, and EDNP in Korea.

Our study has several limitations. First, the cross-sectional nature of our study design indicates that only limited causal associations can be made. Second, several pieces of information were collected from the self-reported questionnaires, so reporting bias cannot be excluded, and comparison with other country results is limited. Third, this study was limited due to a lack of information on participants’ health status, as well as their in-depth knowledge of possible future effects of complex health policies and media regulations related to health.

## Conclusions

We found our nationally sampled population showed strong public support for the taxation and regulation of unhealthy products. Whether an individual tended to support or oppose taxation and regulation was not associated with age, sex, and education. However, individuals that were married, living with a partner, or had one or more children showed support for the regulation of the media and for introducing health taxes. Excessive exposure to the advertising of unhealthy products resulted in individuals being more supportive of its regulation. We recommend introducing regulations and subsidies that would encourage companies to produce healthier products and advertise their health benefits.

## Additional file

Additional file 1: Questionnaire. Questionnaire for: 1) Opinions on alcohol advertisement, smoking scenes portrayed in the media, food-shows, and food advertisement regulations. 2) Exposure to alcohol advertising, smoking scenes portrayed by the media, eating broadcasts, and food advertising and their influence on health habits. 3) Health tax. (DOCX 13 kb)

## Data Availability

The raw data are being kept in the custody of Seoul National University Medical College and are available upon request (contact corresponding author YHY or first author EKK).

## Abbreviations

BMI: Body Mass Index
EDNP: Energy Dense Nutrition Poor
IPTV: Internet Protocol Television
OECD: Organization for Economic Cooperation and Development
SSB: Sugar-Sweetened Beverages
WHO: World Health Organization
