# Supplementary material for: Public support for health taxes and media regulation of harmful products in South Korea
Source: BMC Public Health. 2019 May 30;19:665. doi: 10.1186/s12889-019-7044-2 (PMC6543563; doi:10.1186/s12889-019-7044-2)
Supplement: Supplementary file 1 — Questionnaire. Questionnaire for: 1) Opinions on alcohol advertisement, smoking scenes portrayed in the media, food-shows, and food advertisement regulations. 2) Exposure to alcohol advertising, smoking scenes portrayed by the media, eating broadcasts, and food advertising and their influence on health habits. 3) Health tax. (DOCX 13 kb) [file 12889_2019_7044_MOESM1_ESM.docx]

### Opinions on alcohol advertisement, smoking scenes portrayed in the media, food-shows, and food advertisement regulations

Q1. Some believe that regulatory measures are necessary in the following areas as their portrayal in the mass media has a negative impact on viewers’ health habits. Do you think it is necessary to regulate these areas?

|  | Strongly disagree | Disagree | Agree | Strongly  agree |
| --- | --- | --- | --- | --- |
| 1) Alcohol advertising | **①** | **②** | **③** | **④** |
| 2) Smoking scenes portrayed by the mass media | **①** | **②** | **③** | **④** |
| 3) Eating broadcasts | **①** | **②** | **③** | **④** |
| 4) Food advertisement | **①** | **②** | **③** | **④** |

### Exposure to alcohol advertising, smoking scenes portrayed by the media, eating broadcasts, and food advertising and their influence on health habits

Q2. How often have you been exposed to the following factors during the last week?

|  | Never | Rarely | Occasionally | Always |
| --- | --- | --- | --- | --- |
| 1) Alcohol advertising | **①** | **②** | **③** | **④** |
| 2) Smoking scenes portrayed by the mass media | **①** | **②** | **③** | **④** |
| 3) Eating broadcasts | **①** | **②** | **③** | **④** |
| 4) Food advertisement | **①** | **②** | **③** | **④** |

Q3. How do the following factors affect your health habits?

|  | Never | Rarely | Occasionally | Always |
| --- | --- | --- | --- | --- |
| 1) Alcohol advertising | **①** | **②** | **③** | **④** |
| 2) Smoking scenes portrayed by the mass media | **①** | **②** | **③** | **④** |
| 3) Eating broadcasts | **①** | **②** | **③** | **④** |
| 4) Food advertisement | **①** | **②** | **③** | **④** |

### Health tax

The World Health Organization (WHO) recommended introducing a sugar tax in 2016 on sugar-sweetened beverages (SSB) containing sugars known to be a major cause of chronic diseases. What do you think about the way to impose taxes on health (alcohol, obesity, health tax, etc.) to companies that aggravate alcohol drinking or obesity?

| Strongly disagree | Disagree | Agree | Strongly agree |
| --- | --- | --- | --- |
| **①** | **②** | **③** | **④** |
